# Study on the Shear Strength of Loess Solidified by Guar Gum and Basalt Fiber

**DOI:** 10.3390/ma17133116

**Published:** 2024-06-25

**Authors:** Yu Xi, Mingming Sun, Huanhuan Li, Gang Li, Pengzhou Wang, Li Li

**Affiliations:** 1 Shaanxi Key Laboratory of Safety and Durability of Concrete Structures, Xijing University, Xi’an 710123, China; 2 School of Civil Engineering and Architecture, Ningbo Tech University, Ningbo 315100, China; 3 China Construction Fourth Engineering Division Corp., Ltd., Guangzhou 511400, China

**Keywords:** green materials, fiber length, fiber-reinforcement, model establishment, microscopic mechanism

## Abstract

Loess is widely distributed in the northwest and other regions, and its unique structural forms such as large pores and strong water sensitivity lead to its collapsibility and collapse, which can easily induce slope instability. Guar gum and basalt fiber are natural green materials. For these reasons, this study investigated the solidification of loess by combining guar gum and basalt fiber and analyzed the impact of the guar gum content, fiber length, and fiber content on the soil shearing strength. Using scanning electron microscopy (SEM), the microstructure of loess was examined, revealing the synergistic solidification mechanism of guar gum and basalt fibers. On this basis, a shear strength model was established through regression analysis with fiber length, guar gum content, and fiber content. The results indicate that adding guar gum and basalt fiber increases soil cohesion, as do fiber length, guar gum content, and fiber content. When the fiber length was 12 mm, the fiber content was 1.00%, and the guar gum content was equal to 0.50%, 0.75%, or 1.00%, the peak strength of the solidified loess increased by 82.80%, 85.90%, and 90.40%, respectively. According to the shear strength model, the predicted and test data of the shear strength of solidified loess are evenly distributed on both sides of parallel lines, indicating a good fit. These findings are theoretically significant and provide practical guidance for loess solidification engineering.

## 1. Introduction

Loess is detritus deposited over time, consisting primarily of silt particles and other dust. Therefore, loess has a porous and loose structure, and shallow loess is sensitive to water. Its structure is prone to rapid destruction and strength reduction, resulting in subgrade settlement to a certain extent [1,2,3,4]. Due to Western development, more roads and railways are being built in loess areas, which will face a wide range of loess engineering problems that, if not properly handled, will affect the security and stability of building projects [5,6,7,8]. In the study of the shear strength properties of loess, many scholars both domestically and abroad have conducted shear strength tests on remolded or modified soil. Some scholars use cementitious or fiber materials to reinforce the loess, while others focus on the various factors affecting the shear strength. They have achieved numerous research results. Wu et al. [9] declared that fibers can effectively prevent the appearance and enlargement of obvious cracks in the soil, and improve the shear resistance of solidified soil. Bao et al. [10] found that guar gum has strong absorption capacity for heavy metals in soil, effectively improving soil ecology and providing a good ecological protection role. Jia et al. [11] discovered that adding guar gum considerably improves soil strength and resistance to erosion and that combining guar gum with fiber can make loess particles bind closely, and improve its mechanical properties. Jia et al. [12] discovered that adding basalt fiber strengthens soil, mainly by increasing the cohesion of silty clay to improve the shear strength of soil. Wang et al. [13] pointed out that adding lime and basalt fiber increases soil’s resistance to frost damage, and enhances its unrestricted compressive strength as the fiber quantity increases. The unconfined compressive strength values of samples with a lime content of 3% and 0.5%, 1%, and 1.5% fiber content increased by 39 kPa, 75 kPa, and 106 kPa, respectively. Song et al. [14] analyzed the microscopic mechanism of basalt fiber-fortified red clay using an electron microscope. The fiber combined with soil particles to create a fiber-network structure, preventing soil particles from moving and deforming under external force. At a dry density of 1.45 g/cm^3^, the cohesion of red clay containing 0.2%, 0.3%, and 0.4% basalt fibers increased by 39.7%, 66.7%, and 24.3%, respectively. Xu et al. [15] conducted preliminary research on the shear strength mechanism of fiber-reinforced loess, showing that the shear strength of fiber-fortified soils is significantly greater than that of plain soil. Bulging damage occurs in high-fiber content samples, while shear band damage occurs in non-fibrous or low-fibrous samples. Choobbasti et al. [16] used non-lateral compressive strength and non-consolidated and non-drained triaxial tests to study the non-consolidated strength of soft clay reinforced by nano calcium carbonate at a construction site. With 1.2% nano calcium carbonate, an increase of 50% in unconfined compressive strength was observed. Yang et al. [17] used polypropylene fibers and cement to reinforce loess and found that polypropylene fibers play a bridging role in the soil. When the fiber content is 0.30% and the cement content is 0.60%, the compressive strength without lateral lines reaches 5.13 Mpa. Ple and Lê [18] found that PP fiber increases the ductility of clay, reduces the appearance of cracks, and improves mineral barrier properties. Tang et al. [19] declared that wave-shaped fibers can improve soil strength more than conventional fibers, due to the contact force between the soil particles and the fiber surface. Lian et al. [20] found that adding fibers to the soil markedly increases the shear strength parameters. As the fiber length increases from 9 to 18 mm, the cohesion rises by 23.2%. Patel and Singh [21] pointed out that using glass fiber can increase strength and stabilize the shape of clay; when the fiber content is high, expansion failure can occur.

Most scholars have investigated the mechanical characteristics of fiber-reinforced loess and have proven that fiber has tensile anchoring effects in soil, which can reduce the displacement of soil particles and improve soil strength [22,23,24]. However, studies on loess solidified with a combination of guar gum and basalt fiber are still lacking. This paper analyzes the effects of basalt fiber content, guar gum content, and basalt fiber length on the mechanical characteristics of solidified loess. The microstructure of loess was studied through SEM tests, revealing the synergistic solidification mechanisms of guar gum and basalt fiber. Moreover, a shear strength model was developed, considering the impact of fiber length, guar gum content, and fiber content. This discovery offers a theoretical foundation for the feasibility of loess solidification in engineering construction.

## 2. Materials and Methods

### 2.1. Test Materials

The trial loess was gathered from Chang’an District, Xi’an City, Shaanxi Province, with a sampling depth ranging from 2 to 3 m, totaling 20 kg. During the sampling process, areas with fewer plant roots were selected. The soil sample was then tightly wrapped with cling film to reduce disturbance during transportation. Table 1 lists the loess’s fundamental physical characteristics.

The guar gum used in the experiment was purchased from Zhengzhou Wanbang Chemical Company Co., Ltd. (Zhengzhou, China). It is white in appearance, can form viscous colloids in cold water, and is often used to thicken, solidify, and enhance the texture and stability of food. The basalt fiber was purchased from Shijiazhuang Zhuzhong Technology Co., Ltd. (Shijiazhuang, China). It is a multifunctional fiber material that is bronze in appearance, fluffy after disassembly, and can sink in water. The mechanical and physical properties of the basalt fiber are listed in Table 2 [25].

### 2.2. Sample Preparation

**Figure 1:**
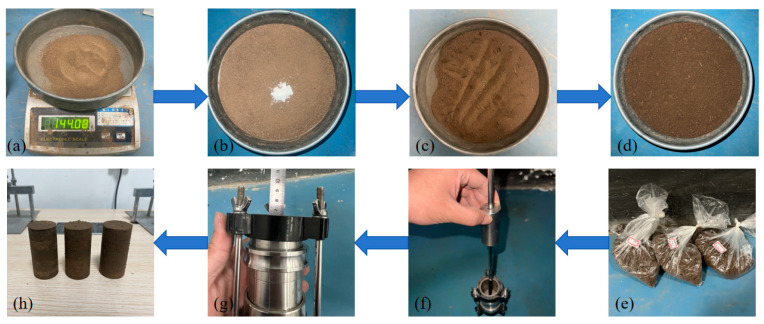
Sample setup flow chart: (**a**) weigh soil; (**b**) add guar gum and stir evenly; (**c**) add fiber and stir evenly; (**d**) add water and stir evenly; (**e**) seal and stand; (**f**) load and prepare sample; (**g**) control the height; (**h**) demold.

According to the standard for geotechnical test methods (GB/T50123-2019) [26], the sample sizes were 39.1 × 80 mm (diameter × height). Before sample preparation, silicone oil was evenly applied to the inner wall of the sample preparation device to prevent soil from sticking to it. The samples were prepared in five layers. To ensure better bonding between layers, scraping treatment was carried out after each layer was compacted. The sample preparation process is shown in Figure 1. A small stirrer was used during the mixing process to achieve a more uniform distribution of guar gum and basalt fibers in the soil.

### 2.3. Test Method

Triaxial shear tests were selected to study the shear behavior of the guar gum-solidified and basalt fiber-fortified loess to more accurately reflect the stress state of the specimen in an actual environment. The UU triaxial shear test was performed using the KTL-LDF 50 static triaxial testing machine. The optimum water content was 20%. Fiber lengths of 4, 8, and 12 mm, fiber contents of 0.20%, 0.60%, and 1.00%, and guar gum contents of 0.50%, 0.75%, and 1.00% were used. The triaxial shear velocity was set to 0.6 mm/min. During the shear process, the termination condition of the test depended on the characteristics of the stress–strain relationship. If the stress–strain relationship showed a softening curve with a peak value, the test was terminated when the peak stress appeared and the axial strain reached 3%~5%. If a strain-hardening curve was observed in the stress–strain relationship, the test was terminated when the axial elongation exceeded 20%. Table 3 shows the test scheme, comprising 120 group experiments.

## 3. Results and Analysis

### 3.1. Analysis of Shear Strength Characteristics of Guar Gum-Solidified Loess

**Figure 2:**
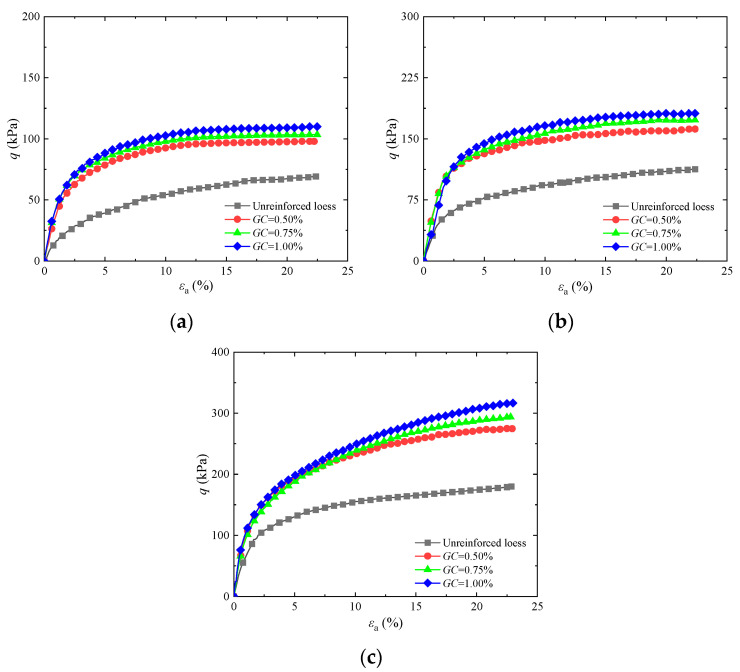
Influence of different contents of guar gum on stress–strain curves of solidified loess: (**a**) *σ*_3_ = 25 kPa; (**b**) *σ*_3_ = 50 kPa; (**c**) *σ*_3_ = 100 kPa.

To research the effects of guar gum content on the shear strength of the solidified soils, Figure 2 shows the curves of guar gum-solidified soil under different confining pressures when the guar gum content was 0.50%, 0.75%, or 1.00%. It can be observed that the stress–strain diagrams of guar gum-solidified and untreated loess under different confining pressures mainly show a trend of hardening. The peak strength of the guar gum-solidified soil is significantly greater than that of the unreinforced loess. The peak strength gradually increased with increasing guar gum content, with its enhancement effect being more pronounced under higher confining pressures. The primary cause of this behavior is that guar gum combined with water in loess produces hydrogel, which can bond soil particles, fill pores, and make the overall structure of soil particles denser. Guar gum creates a higher degree of cementation between soil particles with increased guar gum content, thus improving strength. Additionally, guar gum powder can absorb water in the soil to produce a gel, which reduces the soil water content, thereby solidifying the soil. Therefore, the optimal guar gum content in this test was 1.00%, which is consistent with the findings in [27,28,29].

### 3.2. Analysis of Shear Strength Characteristics of Basalt Fiber-Solidified Loess

**Figure 3:**
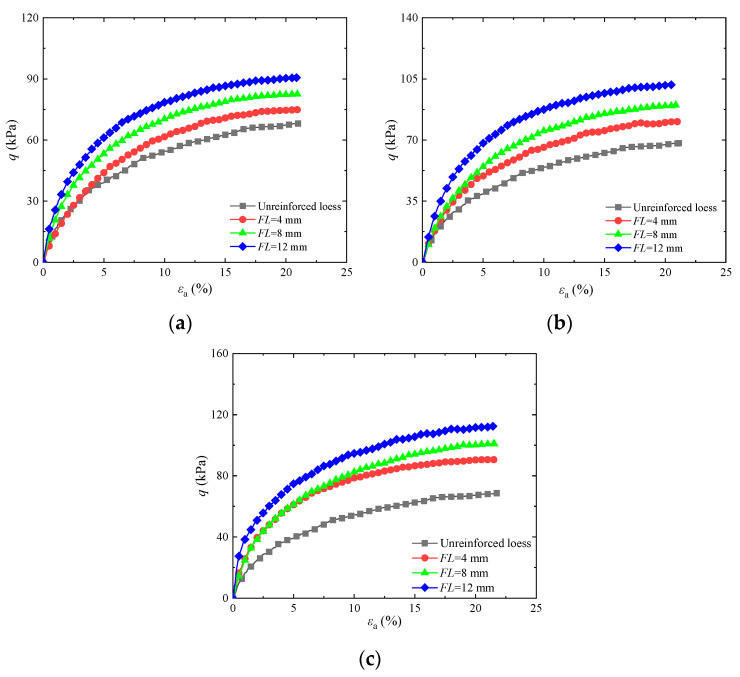
Influence of different basalt fiber lengths on stress–strain diagram of reinforced soil: (**a**) *FC* = 0.20%; (**b**) *FC* = 0.60%; (**c**) *FC* = 1.00%.

To research the impact of fiber length on the shear strength of basalt fiber-fortified loess, Figure 3 displays stress–strain graphs for basalt fiber-fortified loess with different contents under a confining pressure of 25 kPa. The graph shows that the curve of basalt fiber-fortified loess exhibits a hardening trend. The maximum pressure deviation of solidified soil increases with fiber length, and the peak shear strength of reinforced soil is higher than that of untreated soil. Compared to untreated soil, when the length was 4, 8, or 12 mm and the fiber content was equal to 0.20%, the maximum strength increased by 16.50%, 32.70%, and 46.20%, respectively. This is similar to the conclusion of the reference [25]. The primary cause of this behavior is that the fiber and soil particles form a compound structure, enhancing the soil’s shear strength. As fiber length increases, the interfaces between single fibers and soil particles increase, leading to stronger friction and cohesive forces between the fiber and soil. As the scope of action becomes wider, the fixing effect of the fiber in the soil and the interweaving effect of fiber become more significant. Furthermore, under shear stress, the higher tensile properties of the fibers and the interweaving effect further prevent soil failure.

**Figure 4:**
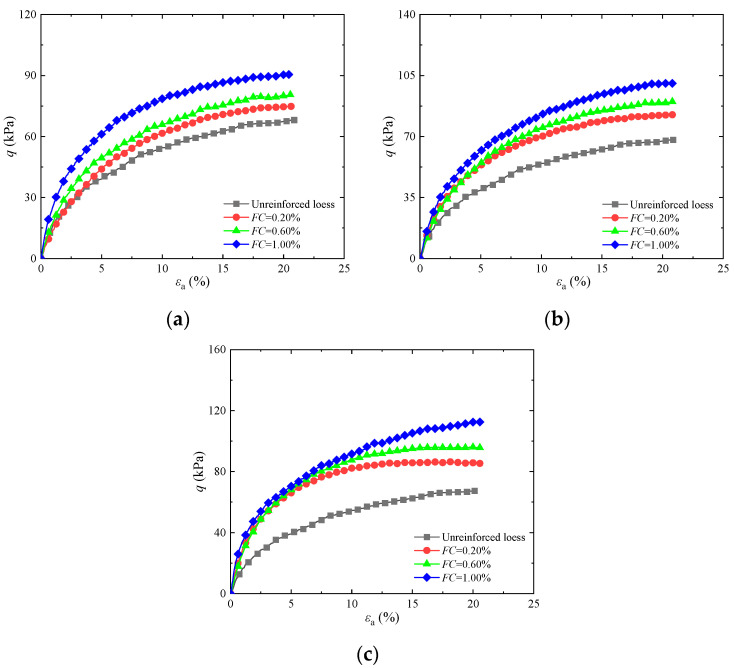
Influence of different basalt fiber contents on stress–strain diagram of reinforced soil: (**a**) *FL* = 4 mm; (**b**) *FL* = 8 mm; (**c**) *FL* = 12 mm.

To analyze how fiber content affects the shear strength of solidified loess, Figure 4 displays stress–strain graphs for basalt fiber-reinforced loess with different fiber lengths under a containment pressure of 25 kPa. The figure shows that the peak force of the fortified loess gradually increases with increasing fiber content. Compared to untreated soil, the peak strength of fortified loess with a fiber length of 4 mm and fiber contents of 0.20%, 0.60%, and 1.00% increased by 13.30%, 20.70%, and 33.60%, respectively. The primary cause of this phenomenon is that fibers form a composite structure with soil particles. By increasing the fiber content, the fibers become more densely distributed among the soil particles, which helps increase the surface area of contact between the fibers and the soil. The anchoring effect of the fibers limits the amount of soil particle deformation that occurs, and the reinforcing effect of the fibers becomes more pronounced, thereby improving the strength of the loess. This is congruent with the conclusion in [30].

### 3.3. Analysis of Shear Strength Characteristics of the Guar Gum and Basalt Fiber-Solidified Loess

**Figure 5:**
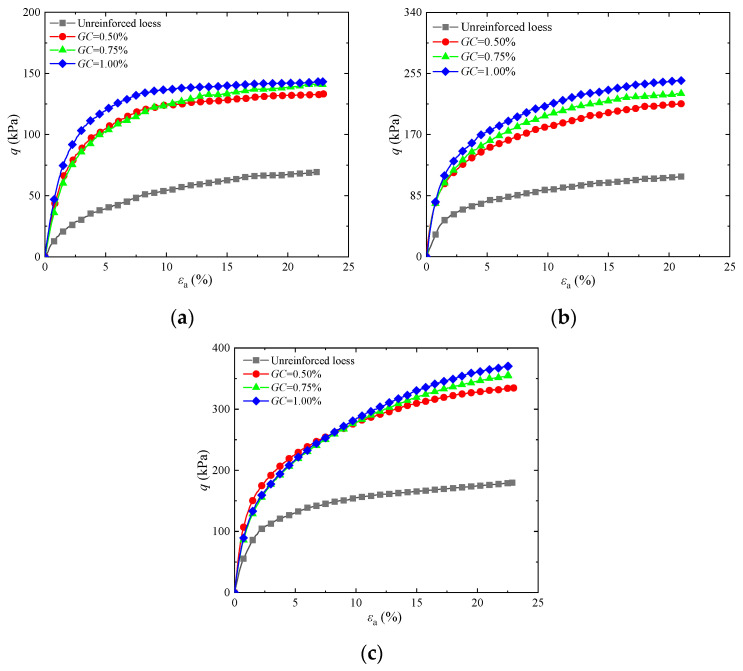
Influence of different guar gum contents on the stress–strain of the loess jointly solidified by guar gum and basalt fiber: (**a**) *σ*_3_ = 25 kPa; (**b**) *σ*_3_ = 50 kPa; (**c**) *σ*_3_ = 100 kPa.

To research the consequences of guar gum content on the shear strength of combined guar gum and basalt fiber-solidified loess, Figure 5a–c display stress–strain graphs for different guar gum contents under various confining pressures when basalt fiber content was 1.00% and the length was 12 mm. With increasing guar gum content, the peak intensity of the solidified loess also progressively rises. Compared to untreated soil, the peak intensity of reinforced loess increased by 82.80%, 85.90%, and 90.40% under a confining pressure of 100 kPa with a 12 mm fiber length, a fiber content of 1.00%, and guar gum contents of 0.50%, 0.75%, and 1.00%, respectively. Compared with lime and basalt fibers, the unconfined compressive strength values of samples with a lime content of 3% and 0.50% and fiber content of 1% and 1.5% increased by 39 kPa, 75 kPa, and 106 kPa, respectively [13]. The peak values of the three stress–strain curves were not significantly different under a confining pressure of 25 kPa. However, under a confining pressure of 100 kPa, all three stress–strain curves showed a significant upward trend, especially the curve with a guar gum content of 1.00%, indicating that the bonding effect of guar gum plays an important role in solidified soil. The primary cause of this behavior is that when guar gum and basalt fiber are added to soil, the guar gum forms a gel after absorbing water, which enhances the adhesion and friction of the soil. The gel combines with the basalt fibers to form a more stable “fiber–gel net,” effectively limiting soil particle movement. This is consistent with the conclusion in [12].

**Figure 6:**
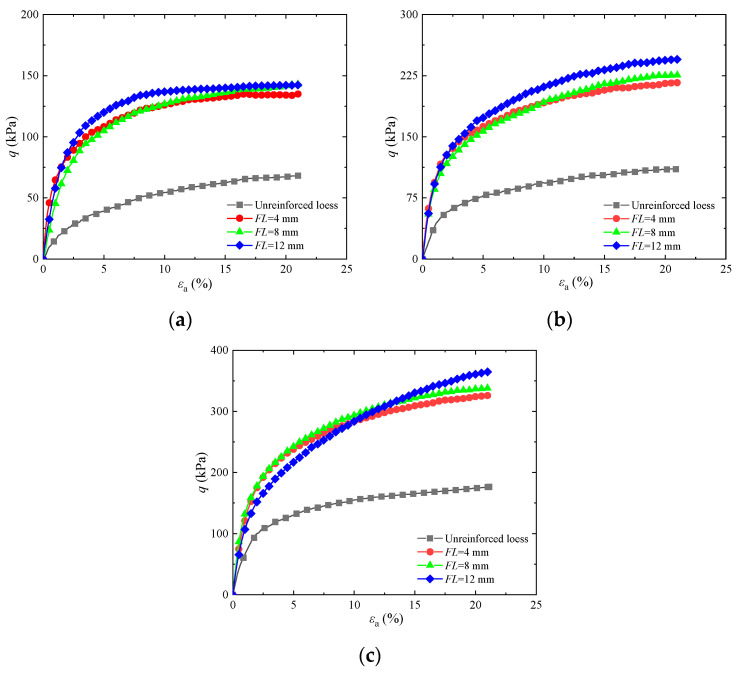
Influence of different fiber lengths on stress–strain of the loess jointly solidified by guar gum and basalt fiber: (**a**) *σ*_3_ = 25 kPa; (**b**) *σ*_3_ = 50 kPa; (**c**) *σ*_3_ = 100 kPa.

Fiber length can affect the shear strength of soil. Figure 6a–c display stress–strain graphs of various fiber lengths at various levels of confinement pressure with a guar gum content of 1.00% and a basalt fiber content of 1.00%. As depicted in the figure, the peak intensity of solidified loess increases as the fiber length gradually increases. Compared to untreated soil, the peak intensity of loess reinforced with a fiber content of 1.00%, a guar gum content of 1.00%, and fiber lengths of 4, 8, and 12 mm increased by 86.7%, 90.7%, and 94.4%, respectively, under a confining pressure of 100 kPa. When the guar gum and basalt fiber contents were 1.00%, all three stress–strain curves showed an upward trend. The stress–strain curves with fiber lengths of 4 and 8 mm overlapped, while the stress–strain curve with a fiber length of 12 mm showed a clear upward trend. This indicates that when the fiber length is 12 mm, the tensile anchoring effect of the fiber is better utilized. The primary cause of this behavior is that the fiber and guar gum form a “fiber–gel net” system. With a constant fiber content, the short fiber lengths reduce the contact area between the fibers and soil particles, resulting in limited coverage of the “fiber–gel net” system. As the fiber length increases, the number of contact points between single fibers and soil particles rises, and the region of interaction between the “fiber–gel net” and soil particles becomes wider, creating a closer connection between the “fiber–gel net” and soil particles.

**Figure 7:**
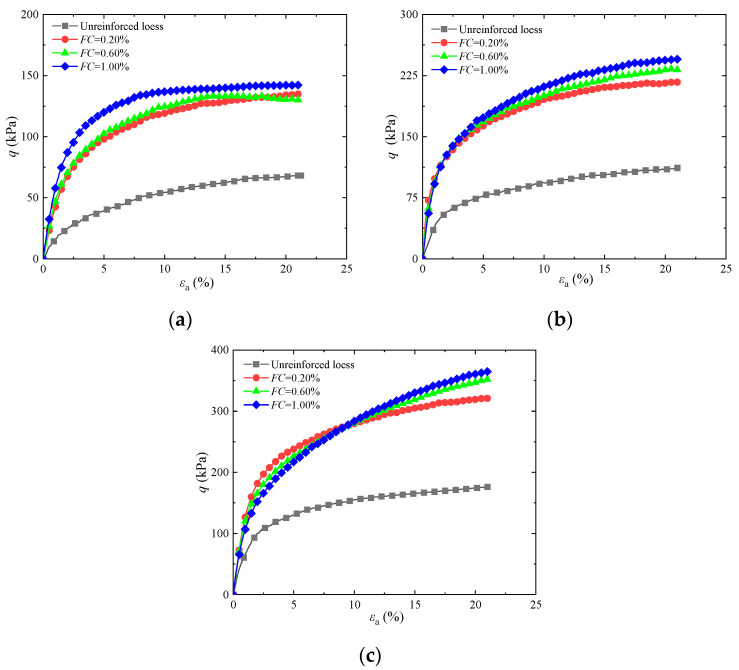
Influence of different fiber contents on stress–strain of the loess jointly solidified by guar gum and basalt fiber: (**a**) *σ*_3_ = 25 kPa; (**b**) *σ*_3_ = 50 kPa; (**c**) *σ*_3_ = 100 kPa.

The fiber content increases the contact surface with soil particles, impacting the shear strength of solidified soil. Figure 7a–c display the stress–strain curves at different levels of confining pressure for varying basalt fiber contents with a guar gum content of 1.00% and a basalt fiber length of 12 mm. As shown in the figure, under constant confining pressure, the peak strength of solidified soil increases with a gradual increase in fiber content. When the confining pressure increases, the peak strength of solidified soil increases, and the difference gradually narrows. Compared to untreated soil, the peak intensity of loess reinforced with a guar gum content of 1.00%, a fiber length of 12 mm, and fiber contents of 0.20%, 0.60%, and 1.00% increased by 82.50%, 88.30%, and 93.40%, respectively, under a confining pressure of 100 kPa. Under a confining pressure of 100 kPa, the peak strength of stress–strain curves with fiber contents of 0.20%, 0.60%, and 1.00%, respectively, showed a significant upward trend, especially with fiber contents of 0.60% and 1.00%. The primary cause of this behavior is that increasing the fiber content enhances the role of the gel network. Due to the excellent tensile performance of the fiber, the gel net can better resist soil damage.

### 3.4. Analysis of the Mechanism of Action for Loess Solidified by Guar Gum and Basalt Fiber

**Figure 8:**
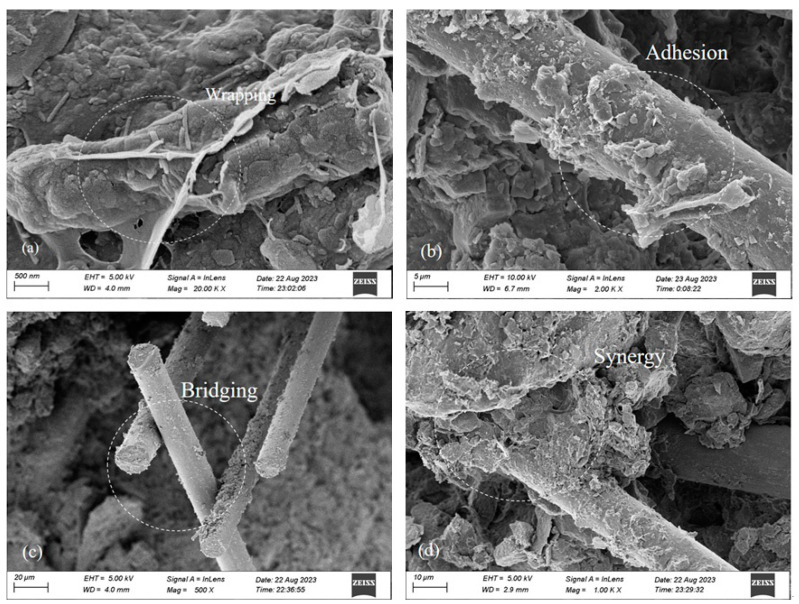
Microscopic structure of the loess jointly solidified by guar gum and basalt fiber: (**a**) wrapping effect; (**b**) adhesion effect; (**c**) bridging effect; (**d**) glue–bar synergy effect.

To study the action mechanism of the guar gum–basalt fiber synergistic solidification of loess, Figure 8 illustrates the microstructure of this solidification process. Figure 8a demonstrates the occurrence of the hydrosol phenomenon when guar gum interacts with water, effectively coating soil particles and preventing them from moving between particles, thereby improving soil strength. Figure 8b,c show guar gum aqueous solution attached to the fiber surface, with fibers overlapping to form a fiber gel–gum net structure. Single fibers can bridge together, limiting the relative sliding of soil particles through bending, embedding, and bridging. Multiple fibers can form a supporting network, creating a fiber network structure; soil particles interlock due to the network architecture, which restricts the deformation and displacement of soil particles. This is consistent with the conclusion in [14]. Guar gum attached to fibers strengthens their bridging effect and forms a “fiber–gel net” to stabilize the bridging between the fibers. Figure 8d presents how guar gum and basalt fiber work together; basalt fiber forms an anchorage zone in the soil to tightly lock soil particles, while guar gum wraps soil particles around it and stabilizes the interaction among multiple basalt fibers, forming a dual stability effect. When soil is stressed, the internal fibers endure tensile distortion of the soil, and guar gum hydrates fill some of the voids within the soil. This increases the soil compactness and enhances the contact area between the fibers and soil and the interaction force between interfaces, which results in better mechanical properties for fiber-reinforced soil.

## 4. Modeling of Shear Strength Model of Guar Gum and Basalt Fiber-Solidified Loess

### 4.1. Model Establishment

**Figure 9:**
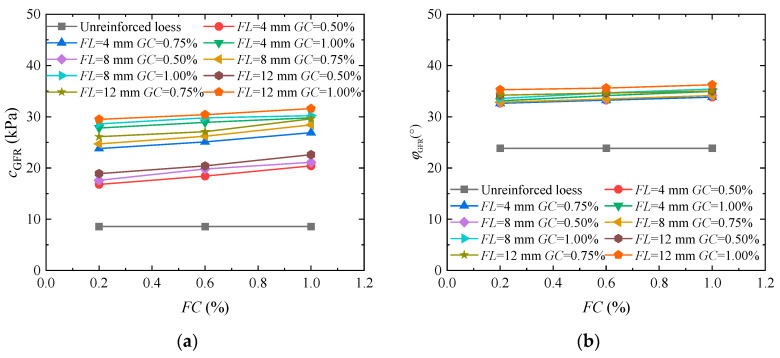
Cohesion and angle analysis of the internal friction indicators of loess jointly solidified by guar gum and basalt fiber: (**a**) cohesion; (**b**) angle of internal friction.

To analyze the impact of guar gum and basalt fiber-solidified loess on the shear strength index, Figure 9a shows the cohesion curve when the fiber length was equal to 4, 8, or 12 mm; the guar gum content was 0.50%, 0.75%, or 1.00%; and the fiber content was 0.20%, 0.60%, or 1.00%. The figure demonstrates a rapid increase in soil cohesion upon the simultaneous addition of basalt fiber and guar gum. When the fiber length was 4 mm and the guar gum content was 0.50%, there was not much of a difference in cohesion compared to a fiber length of 8 mm and a guar gum content of 0.50%. However, when the fiber length was 4 mm and the guar gum content was 0.50%, there was a significant difference in cohesion compared to a fiber length of 4 mm and guar gum content of 0.75%. This is due to the increase in guar gum content, which generates increased mucus production as a result of the reaction between guar gum and water, increasing the bonding area and better stabilizing the movement between fibers and soil particles. This indicates that an increase in guar gum results in a more prominent role of the “fiber–gel mesh” in wrapping soil. The internal friction angle diagram for solidified loess containing guar gum and basalt fibers is displayed in Figure 9b. The figure shows that the soil’s internal friction angle rises rapidly when guar gum and basalt fiber are added together. Despite increases in fiber content, fiber length, and guar gum content, there is little change in the soil’s angle of internal friction because the “fiber–gel net” formed by the combination of fiber and guar gum fills the soil pores and stabilizes the displacement of soil particles.

The transformation system of guar gum and basalt fiber bear load together. According to the results presented above, the hypothesis is that the *c*_GFR_ cohesion of reinforced soil is a function of *GC*, *FC*, *FL,* and d [25].

(1) $$c_{GFR}=f\left(\frac{FL\cdot FC}{90d+FL\cdot FC}+3GC\right)c_{0}$$

where *c*_0_ and *c*_GFR_ are the initial and strengthened cohesion of loess, *FL* is the fiber length, *FC* is the fiber content, d is the fiber diameter, and *GC* is the guar gum content.

Equation (1) can be written as:

(2) $$\frac{c_{GFR}}{c_{0}}=f\left(\frac{FL\cdot FC}{90d+FL\cdot FC}+3GC\right)$$

The curves of *c*_GFR_/*c*_0_ and (*FL*·*FC*/90d) + 3*GC* are drawn by the experimental data. It was discovered that *c*_GFR_/*c*_0_ increases by increasing (*FL·FC*/90d) + 3*GC*, and the relationship is linear.

This is given by Equation (2):

(3) $$c_{GFR}=\left[a+b\left(\frac{FL\cdot FC}{90d+FL\cdot FC}+3GC\right)\right]c_{0}$$

where the fitting curve’s slant and intercept are represented by the doping numbers *a* and *b*.

It is shown in Figure 8b that as the fiber length, guar gum, and fiber content increase, the angle of internal friction of the solidified loess changes little. Therefore, the mean value of the internal angle of friction of the solidified loess can be taken:

(4) $$\tan\varphi_{GFR}=34.2{}^{\circ}$$

The angle of internal friction of solidified loess can be obtained by combining Equation (1) with test Equation (4) according to Mohr–Coulomb theory tan*φ_GFR_*.

(5) $$\tau_{GFR}=c_{GFR}+\tan\varphi_{GFR}\sigma$$

In the equation, the shear strength of solidified loess is calculated, and *σ* is the stress $\tau_{GFR}$.

Inserting Equations (3) and (4) into Equation (5) gives:

(6) $$\tau_{GFR}=\left[a+b\left(\frac{FL\cdot FC}{90d+FL\cdot FC}+3GC\right)\right]c_{0}+\tan\varphi_{GFR}\sigma$$

According to Equation (6), when the parameters *c*_0_, *φ*_0_, *GC*, *FL*, *FC*, and d are known, the fitting method can be used to obtain the unknown parameters *a* and *b*.

### 4.2. Model Parameter Fitting

**Figure 10:**
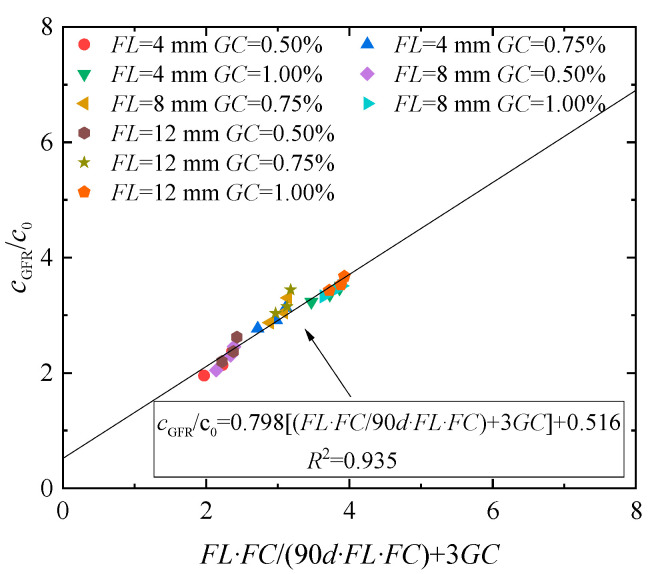
The fitting curve of the cohesion force of the loess jointly solidified by guar gum and basalt fiber.

Figure 10 presents the fitting effect of the solidified loess with guar gum contents of 0.50%, 0.75%, and 1.00%, fiber contents of 0.20%, 0.60%, and 1.00%, and fiber lengths of 4, 8, and 12 mm. It can be seen that the value of *c*_GFR_/*c*_0_ increases linearly with increasing *FL*·*FC*/(90d + *FL*·*FC*), and the fitting effect is positively correlated with a good linear relationship. As shown in Figure 10, the cohesion of solidified soil has a good relationship with fiber length, fiber content, and guar gum content. Through regression analysis, the *a* and *b* parameter values were obtained, and the determinable coefficient of the fitting curve reached 0.935; this indicates a linear relationship between the horizontal and vertical axes of the model, which can be represented by a linear equation. The shear strength index parameters of the guar gum and basalt fiber-solidified loess were determined as *a* = 0.516 and *b* = 0.798.

### 4.3. Model Verification

**Figure 11:**
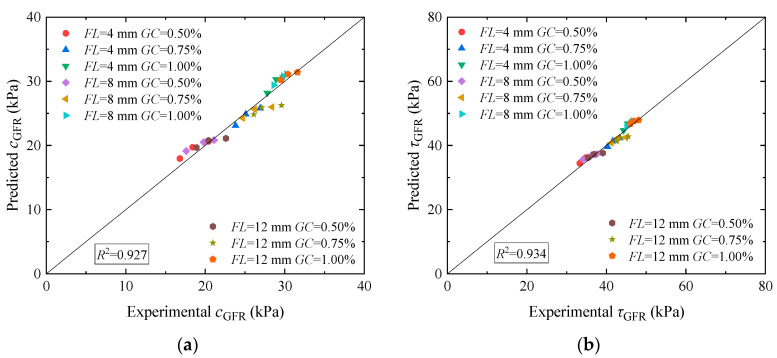
Comparison of the shear strength between the predicted and test results of loess jointly solidified by guar gum and basalt fiber: (**a**) cohesion prediction; (**b**) shear strength prediction.

To verify model reliability, the *c*_0_, *GC*, *FL*, and *FC* of the loess, along with the shear strength index parameters a and b of the guar gum and basalt fiber-solidified loess, were brought into Equation (3) to determine the cohesion of guar gum and basalt fiber-solidified loess. The shear strength of the guar gum and basalt fiber-solidified loess was calculated by bringing parameters *a* and *b* into Equation (6). Figure 11a compares the predicted cohesive force of the solidified loess with that of the tested loess. It can be found that the data of the predicted cohesive force and tested cohesive force are evenly distributed on both sides of parallel lines. Figure 11b compares the predicted and tested shear strength of the solidified loess. It can be seen that the data of the predicted and tested shear strength are more concentrated on both sides of the parallel lines, indicating that the predicted shear strength of the solidified loess agrees well with the test results. By comparing the cohesive force and shear strength, the forecast values align with the experimental values, which shows that the model is appropriate for forecasting the cohesive force and shear strength of guar gum and basalt fiber-solidified loess.

## 5. Conclusions

The impact of confining pressure, fiber length, fiber content, and guar gum content on the shear strength of solidified loess was analyzed through triaxial unconsolidated and undrained shear tests. On this basis, a shear strength model was established through regression analysis incorporating fiber length, guar gum content, and fiber content. The principal results are as follows:

(1) The shearing strength of the loess was improved by adding guar gum into the loess, and the peak strength gradually rose as the guar gum content increased. Compared with untreated soil, the peak strength increased to 107.98 kPa, and the optimal content was 1.00%.

(2) The fiber-reinforced loess showed strain hardening and the peak strength of the fiber-reinforced soil increased with increasing fiber length. Compared to untreated soil, the peak strength of solidified loess with a fiber content of 0.20% and fiber lengths of 4, 8, and 12 mm increased by 16.50%, 32.70%, and 46.20%, respectively.

(3) Combining guar gum and basalt fiber effectively improved the shear strength of the solidified loess. When the fiber length was 12 mm, the fiber content was 1.00%, and the guar gum content was equal to 0.50%, 0.75%, or 1.00%, the peak strength of the solidified loess increased by 82.80%, 85.90%, and 90.40%, respectively.

(4) Through regression analysis, the determined coefficients of fitting curves in Figure 11a,b reached 0.927 and 0.934. The predicted results are relatively in line with the outcomes of the experiment. This model could be utilized to forecast the shear strength of guar gum and basalt fiber-solidified loess.

(5) In future studies, dry–wet and freeze–thaw cycle tests will be conducted to study the strength of solidified soil in different environments, and model tests will be conducted in conjunction with slope treatment engineering to study the erosion resistance of solidified loess.

## Figures and Tables

**Table 1:** Loess’s fundamental physical characteristics.

Water Content (%)	Dry Density (g·cm^−3^)	Specific Gravity Gs	Initial Porosity Ratio	Plasticity Limit (%)	Liquid Limit (%)	Plasticity Index
14.50	1.24	2.67	0.73	18.60	33.80	15.20

**Table 2:** Physical and mechanical properties of basalt fiber.

Density/ (g·cm^−3^)	Diameter (μm)	Tensile Strength (MPa)	Elastic Modulus (GPa)	Elongation at Break (%)	Acid and Alkali Resistance
2.65	10	3500~4500	100	2.20	Strong

**Table 3:** Triaxial shear scheme.

Confining Pressure *σ*_3_ (kPa)	Water Content *ω* (%)	Fiber Length *FL* (mm)	Fiber Content *FC* (%)	Guar Gum Dosage *GC* (%)
25	20	0	0	0, 0.50, 0.75, 1.00
		4	0.20, 0.60, 1.00	0, 0.50, 0.75, 1.00
		8	0.20, 0.60, 1.00	0, 0.50, 0.75, 1.00
		12	0.20,0.60,1.00	0, 0.50, 0.75, 1.00
50	20	0	0	0, 0.50, 0.75, 1.00
		4	0.20, 0.60, 1.00	0, 0.50, 0.75, 1.00
		8	0.20, 0.60, 1.00	0, 0.50, 0.75, 1.00
		12	0.20, 0.60, 1.00	0, 0.50, 0.75, 1.00
100	20	0	0	0, 0.50, 0.75, 1.00
		4	0.20, 0.60, 1.00	0, 0.50, 0.75, 1.00
		8	0.20, 0.60, 1.00	0, 0.50, 0.75, 1.00
		12	0.20, 0.60, 1.00	0, 0.50, 0.75, 1.00

## Data Availability

All data are contained within the article.

## References

[1] Luo L., Wang X., Xue C., Wang D., Lian B. (2022). Laboratory experiments and numerical simulation study of composite-material-modified loess improving high-speed railway subgrade. Polymers.

[2] Li Y., Shi W., Aydin A., Beroya-Eitner M.A., Gao G. (2020). Loess genesis and worldwide distribution. Earth Sci. Rev..

[3] Tabarsa A., Latifi N., Meehan C.L., Manahiloh K.N. (2018). Laboratory investigation and field evaluation of loess improvement using nanoclay—A sustainable material for construction. Constr. Build. Mater..

[4] Zhao M., Guo W., Chen L.Y., Wang S. (2019). Experiment on the frost resistance of Modified Phospho Gypsum: A case used to Improve Baozhong Railway Subgrade loess. J. Mt. Sci..

[5] Smalley I.J., Jefferson I.F., Dijkstra T.A., Derbyshire E. (2001). Some major events in the development of the scientific study of loess. Earth Sci. Rev..

[6] Chen G., Zhang Y., Zeng R., Yang Z., Chen X., Zhao F., Meng X. (2018). Detection of land subsidence associated with land creation and rapid urbanization in the Chinese loess plateau using time series insar: A case study of Lanzhou new district. Remote Sens..

[7] Lv Y., Deng L., Fan W. (2021). Loess collapsibility characteristics of railway engineering sites using large-scale trial immersion pit experiments. Bull. Eng. Geol. Environ..

[8] Khattak M.J., Alrashidi M. (2006). Durability and mechanistic characteristics of fiber reinforced soil–cement mixtures. Int. J. Pavement Eng. Int..

[9] Wu Z., Xu J., Chen H., Shao L., Zhou X., Wang S. (2022). Shear strength and mesoscopic characteristics of basalt fiber–reinforced loess after dry–wet cycles. J. Mater. Civ. Eng..

[10] Bao H., Liu C., Lan H., Yan C., Li L., Zheng H., Dong Z. (2022). Time-dependency deterioration of polypropylene fiber reinforced soil and guar gum mixed soil in loess cut-slope protecting. Eng. Geol..

[11] Jia Z., Yan C., Li B., Shi Y., Lan H., Xu J., Bao H. (2022). Experimental study on erosion resistance and ecological slope protection of guar gum-treated fiber-reinforcement loess. Chin. J. Geotech. Eng..

[12] Jia Y., Zhang J., Wang X., Ding Y., Chen X., Liu T. (2022). Experimental study on mechanical properties of basalt fiber-reinforced silty clay. J. Cent. South Univ..

[13] Wang W., Cao G., Li Y., Zhou Y., Lu T., Zheng B., Geng W. (2022). Effects of freeze–thaw cycles on strength and wave velocity of lime-stabilized basalt fiber-reinforced loess. Polymers.

[14] Song Y., Geng Y., Dong S., Ding S., Xu K., Yan R., Liu F. (2023). Study on Mechanical Properties and Microstructure of Basalt Fiber-Modified Red Clay. Sustainability.

[15] Xu J., Wu Z., Chen H., Shao L., Zhou X., Wang S. (2021). Study on strength behavior of basalt fiber-reinforced loess by digital image technology (DIT) and scanning electron microscope (SEM). Arab. J. Sci. Eng..

[16] Choobbasti A.J., Samakoosh M.A., Kutanaei S.S. (2019). Mechanical properties soil stabilized with nano calcium carbonate and reinforced with carpet waste fibers. Constr. Build. Mater..

[17] Yang B., Weng X., Liu J., Kou Y., Jiang L., Li H., Yan X. (2017). Strength characteristics of modified polypropylene fiber and cement-reinforced loess. J. Cent. South Univ..

[18] Ple O., Lê T.N.H. (2012). Effect of polypropylene fiber-reinforcement on the mechanical behavior of silty clay. Geotext. Geomembr..

[19] Tang C., Li J., Wang D., Shi B. (2016). Investigation on the interfacial mechanical behavior of wave-shaped fiber reinforced soil by pullout test. Geotext. Geomembr..

[20] Lian B., Peng J., Zhan H., Cui X. (2020). Effect of randomly distributed fibre on triaxial shear behavior of loess. Bull. Eng. Geol. Environ..

[21] Patel S.K., Singh B. (2017). Strength and deformation behavior of fiber-reinforced cohesive soil under varying moisture and compaction states. Geotech. Geol. Eng..

[22] Wang X., Liu K., Lian B. (2021). Experimental study on ring shear properties of fiber-reinforced loess. Bull. Eng. Geol. Environ..

[23] Xu J., Wu Z., Chen H., Shao L., Zhou X., Wang S. (2022). Influence of dry-wet cycles on the strength behavior of basalt-fiber reinforced loess. Eng. Geol..

[24] Xue Z., Cheng W., Wang L., Song G. (2021). Improvement of the shearing behaviour of loess using recycled straw fiber reinforcement. KSCE J. Civ. Eng..

[25] Chen C., Li G., Liu J., Xi Y., Nan J. (2023). Shear strength characteristics of basalt fiber-reinforced loess. Sci. Rep..

[26] (2019). Standard for Geotechnical Testing Method.

[27] Jia Z., Yan C., Li B., Bao H., Lan H., Liang Z., Ren J. (2023). Performance test and effect evaluation of guar gum-stabilized loess as a sustainable slope protection material. J. Clean. Prod..

[28] Ma Y., Bao H., Yan C., Lan H., Peng J., Zheng H., Liu C. (2023). Mechanical properties and microstructure evolution of two ecological slope-protection materials under dry-wet cycles. J. Clean. Prod..

[29] Sujatha E.R., Saisree S. (2019). Geotechnical behaviour of guar gum-treated soil. Soils Found..

[30] Wei H., Zhao T., Meng Q., Wang X., He J. (2018). Experimental evaluation of the shear behavior of fiber-reinforced calcareous sands. Int. J. Geomech..

